# The effects of resistance training on cardiovascular factors and anti-inflammation in diabetic rats

**DOI:** 10.1016/j.heliyon.2024.e37081

**Published:** 2024-08-28

**Authors:** Jin Yoo, Jinsu Hwang, Jiyun Choi, Mahesh Ramalingam, Haewon Jeong, Sujeong Jang, Han-Seong Jeong, Daeyeol Kim

**Affiliations:** a Department of Physical Education, Chonnam National University, Gwangju, 61186, Republic of Korea; b Department of Physiology, Chonnam National University Medical School, Hwasun-gun, Jeollanamdo, 58128, Republic of Korea; c StemCell Bio Incorporated, Hwasun-gun, Jeollanamdo, 58128, Republic of Korea

**Keywords:** Diabetes, Resistance exercise, Cardiovascular response, Endothelia, Inflammation

## Abstract

Diabetes induces a range of macrovascular and microvascular changes, which lead to significant clinical complications. Although many studies have tried to solve the diabetic problem using drugs, it remains unclear. In this study, we investigated whether resistance exercise affects cardiovascular factors and inflammatory markers in diabetes. The study subjected Otsuka Long-Evans Tokushima Fatty (OLETF) rats, which have genetically induced diabetes mellitus, to a resistance exercise program for 12 weeks and assessed the levels of cardiovascular factors and inflammatory markers using western blotting analysis, ELISA, and immunohistochemistry. During the training period, OLETF + exercise (EX) group exhibited lower body weight and reduced glucose levels when compared with OLETF group. Western blotting analysis, ELISA, and immunohistochemistry revealed that the levels of PAI-1, VACM-1, ICAM-1, E-selectin, TGF-β, CRP, IL-6, and TNF-α were decreased in OLETF + EX group when compared with the OLETF group. Moreover, the anti-inflammatory markers, IL-4 and IL-10, were highly expressed after exercise. Therefore, these results indicate that exercise may influence the regulation of cardiovascular factors and inflammatory markers, as well as help patients with metabolic syndromes regulate inflammation and cardiovascular function.

## Highlights

• Resistance training reduces blood glucose levels.
• Exercise lowers the levels of cardiovascular factors.
• Exercise regulates inflammation.
• Resistance training elevates anti-inflammatory factors.

## 1 Introduction

Diabetes, a chronic metabolic disease that affects an individual's physical, social, and mental well-being, leads to chronic hyperglycemia because of impaired response to insulin and/or impaired insulin secretion [[1], [2], [3]]. Insulin plays a key role in lowering blood glucose levels by inducing accelerated blood glucose transport into muscle cells and glucose uptake, leading to normal blood glucose levels during hyperglycemic reactions to emergencies or stress [4]. There are two main types of diabetes mellitus, type 1 diabetes mellitus (T1DM) and type 2 diabetes mellitus (T2DM) [5]. T1DM is a caused by insulin deficiency after the loss of pancreatic β-cells [[6], [7], [8]], whereas T2DM is characterized by insulin resistance and progressive β-cell dysfunction [9].

Cardiovascular complications are leading causes of death and disability in patients with diabetes [10,11]. When compared with people without diabetes, patients with T2DM have a disproportionately higher rate of cardiovascular diseases (CVD)-associated morbidity and mortality [[11], [12], [13]]. Epigenetic factors, reactive oxygen species, glucolipotoxicity, activation of various transcription-mediated pathways, and increased levels of various pro-inflammatory cytokines, contribute to insulin resistance and the pathogenesis of T2DM [14]. Individuals with metabolic syndrome exhibit simultaneously combined pro- and anti-inflammatory conditions which more probably can lead to cardiovascular diseases progression, T2DM, and some types of cancer, such as pancreatic cancer [15], prostate cancer [16], gastrointestinal stromal tumors [17], and gastric cancer [18,19]. Insulin resistance has been shown to substantially correlate with the plasma levels of plasminogen activator inhibitor 1 (PAI-1) [20], which is an important risk factor for CVD [[21], [22], [23]]. T2DM and CVD may be associated with elevated levels of PAI-1 production and circulation [23,24]. PAI-1 can control the levels of various factors, including growth factors (e.g., transforming growth factors (TGF)-β), hormones (e.g., insulin), inflammatory cytokines (e.g., tumor necrosis factor (TNF)-α and interleukin (IL)-6), and endotoxins [25,26]. Increased levels of C-reactive protein (CRP), TNF-α, and IL-6 have been observed in diabetes [[27], [28], [29]]. In addition, CRP levels are raised by several pro-inflammatory cytokines, including IL-6 and TNF-α, which increase oxidative stress and decrease eNOS phosphorylation, thereby enhancing endothelial dysfunction in patient with T2DM [30,31].

To help patients with T2DM reach and maintain their therapeutic goals and improve their quality of life, physical activity is essential, along with medication, diet control, bariatric surgery, and exercise [32,33]. Exercise therapy includes aerobic exercise, resistance exercise, high-intensity interval training, and combined exercise [[34], [35]]. Patients with diabetes should undergo moderate- or vigorous-intensity aerobic training for >150 and > 75 min per week, respectively [[36], [37]]. However, for people with severe obesity, arthritis, physical disabilities, or diabetes complications, walking, even for 20–30 min, can be difficult, uncomfortable, or painful [38]. Recent studies show that resistance training may be able to prevent, treat, and even reverse several chronic conditions [39]. It can result in positive muscle mass changes and enhance glucose storage and blood glucose clearance [40]. Furthermore, resistance training may increase bone mineral density, lean body mass, and muscular strength, which may improve glycemic management and prevent osteoporosis and sarcopenia [38]. Previous studies indicate that performing resistance training may efficiently influence middle-aged and older people to reduce insulin sensitivity and prevent T2DM [41,42].

Several studies indicate that resistance exercise may be especially beneficial for people with T2DM because they increase insulin activity [43], while reducing the risk of low-grade inflammatory disorders like atherosclerosis, obesity, and insulin resistance [44]. In addition, exercise suppresses TGF-β production and cellular aging, thereby preventing and delaying CVD [45]. General exercise programs increase the number of smooth muscle and endothelial cells, dilate arterial diameter, and expand aortic vessels [46].

However, few studies have investigated the mechanisms underlying the relationship between cardiovascular factors and anti-inflammation following resistance training. This study investigated the effects of 12-week resistance training on cardiovascular and inflammation responses in a rat model of T2DM. We subjected the rats’ heart sample to western blotting analysis, ELISA, and immunohistochemistry analyses. This study aimed to suggest an enhanced resistance training program for patients with diabetes.

## 2 Materials and methods

### 2.1 Animals

Animal experiment protocols were approved by the Institutional Animal Care and Use Committee (CNU IACUC-YB-2020-95) of Chonnam National University, Republic of Korea. Every effort was made to lessen the suffering of the animals. Male Otsuka Long-Evans Tokushima Fatty (OLETF) rats (n = 24), an obese genetic rat model of T2DM, and Long-Evans Tokushima Otsuka (LETO) rats (n = 14), a genetic control group, aged four weeks, were purchased from Central Lab. Animal, Inc. (Seoul, Republic of Korea). Glucose level measurements at 19 weeks of age confirmed that the OLETF group had diabetes. The LETO rats formed the control group, whereas the OLETF rats were randomly assigned to the diabetes group (n = 11) and diabetes with resistance exercise group (OLETF + EX, n = 13). The OLETF + EX group was raised for another 15 weeks, with exercise from the age of 19 weeks, when diabetes was detected. The rats were housed at 24 °C, with a 12-h light/dark cycle and free access to food and water.

### 2.2 Resistance exercise

The rats underwent resistance exercise three days a week, eight times a day, for 12 weeks on a climbing ladder (length: 110 cm, space between rungs: 1.6 cm, width: 20 cm, inclination: 80°) modified from a previous study [47]. Before resistance exercises, the rats underwent pre-adaptation training three days a week, three times a day, for two weeks without loads. In the first and second weeks after starting resistance training, they underwent exercise without loads for three days a week, five times a day. In weeks three, four, and five, and weeks six to seven, loads equivalent to 50 %, 75 %, 90 %, and 100 % of the rats’ body weights were attached to their tails three days a week, eight times a day. From week eight, the rats underwent gradual resistance exercises with loads equivalent to 50 %, 75 %, 90 %, and 100 % of their body weights, four times and four to eight times, and eventually with an additional 30 g to the respective rat weights. Without loads, the rats were allowed to rest for 1 min between each set of exercises. With weight exercises, they were allowed to rest for 2 min between each set of exercises. The load exercise steps are listed in Table 1.

**Table 1:** Resistance exercise program.

Weeks	Resistance exercise program	Times/Period
1–2	Body weight	3 days/week
3	+50 % of body weight	
4	+75 % of body weight	
5	+90 % of body weight	
6–7	+100 % of body weight	
8–12	incremental load exercise +50 %, 75 %, 90 %, 100 %, 100 % + 30g of body weight	

### 2.3 Measurement of body weight and blood glucose

The rats were monitored by measuring their body weights once a week using a weighing scale (FX-2000i, A&D Company, Limited, Tokyo, Japan). Blood glucose levels were monitored weekly after exercise and fasting for 16 h by restraining the rats using rat holders and drawing blood samples from the tail vein. Blood glucose was measured using an ACCU-CHEK glucometer (Active, Roche, Basel, Switzerland).

### 2.4 Intraperitoneal glucose tolerance test

In this study, we confirmed that the rats had diabetes using an intraperitoneal glucose tolerance test (IPGTT) that was modified from a previous study [48]. Briefly, after 16 h of fasting, we intraperitoneally injected the rats with a glucose solution (2 g/kg). Next, they were restrained in rat holders for tail vein blood collection, followed by blood glucose measurement after 30, 60, and 90 min as described above.

### 2.5 Tissue preparation

Animals were anesthetized using sodium pentobarbital (75 mg/kg), transcardially perfused with phosphate-buffered saline (PBS, BIOSESANG, Yongin, Republic of Korea) or fixed with fresh paraformaldehyde (PFA, BIOSESANG) in 0.1 M phosphate buffer (pH 7.4, Sigma-Aldrich, Burlington, USA). For western blotting analysis, PBS-perfused hearts were stored at −80 °C. For immunohistochemistry, the hearts were harvested, fixed in PFA for 24 h, and then stored in 30 % sucrose for three days [49].

### 2.6 Western blotting analysis

Protein concentrations were determined using the BCA Protein Assay Kit (Thermo Scientific, Rockford, IL, USA). Equal protein amounts (15 μg/10 μl) were separated on 7 or 10 % SDS-polyacrylamide (Bio-Rad, Hercules, USA) and then transferred onto polyvinylidene difluoride membranes (Merck Millipore, Burlington, USA). The membranes were then blocked using 5 % skimmed milk (BD, Franklin Lakes, USA) in PBS with 0.1 % Tween-20 (T-PBS) for 60 min and incubated overnight at 4 °C with anti-PAI-1 (1:1000, ab222754, abcam, Cambridge, UK), anti-VCAM-1 (1:1000, ab134047, abcam), anti-ICAM-1 (1:1000, ab171123, abcam), anti-E-selectin (1:1000, sc-137054, Santa Cruz Biotechnology, Inc., Texas, USA), anti-TGF- β (1:1000, ab215715, abcam), anti-CRP (1:1000, ab259862, abcam), anti-TNF-a (1:1000, ab205587, abcam), IL-6 (1:1000, ab9324, abcam), anti-IL-4 (1:1000, sc-53084, Santa Cruz Biotechnology), and anti-IL-10 (1:1000, A2171-100, ABclonal Science, Inc., Hubei, China). The membranes were then washed for 30 min in T-PBS and incubated in an HRP-conjugated secondary antibody (Cell Signaling Technology, Danvers, USA) in a blocking buffer. They were conducted by signal development using the Immobilon Crescendo Western HRP Substrate (Millipore, Burlington, USA). The signals were then imaged on a LAS 4000 system (GE Healthcare, Japan) [50].

### 2.7 ELISA

The levels of cardiovascular factors and immune reactions were measeured using ELISA kits (Elabscience, Wuhan, China). Following the protocol, 100 μl of tissue lysates were incubated with 100 μl biotinylated detection antibodies against PAI-1, VCAM-1, ICAM-1, E-selection, TGF-β, CRP, TNF-α, IL-6, IL-4, and IL-10, at 37 °C for 90 min. After substrate addition, the protein signal was measured at a wavelength of 450 nm on a microplate reader (BioTek, Winooski, USA) according to the manufacturer's specifications [51].

### 2.8 Immunohistochemistry

For immunohistochemical analysis, tissues were cryo-sectioned onto slides using a DE/CM1860 cryo-microtome (Leica Biosystems, Nussloch, Germany). They were then incubated with primary antibodies against PAI-1 (1:100, abcam), VCAM-1 (1:400, abcam), ICAM-1 (1:100, abcam), E-selectin (1:500, Santa Cruz Biotechnology), TGF-β (1:500, abcam), CRP (1:2200, abcam), TNF-α (1:100, abcam), IL-6 (1:1000, abcam), IL-4 (1:50, Santa Cruz Biotechnology), and IL-10 (1:500, ABclonal Science, Inc., Woburn, USA) diluted in 10 % normal goat serum (NGS, Vector Laboratories, Newark, USA), at room temperature for 1.5 h. Next, they were incubated with biotinylated anti-rabbit secondary antibody (1:500, Santa Cruz Biotechnology) and the signal was developed using a Vector Elite ABC kit (1:100, Vector Laboratories). The antibody-biotin-avidin-peroxidase complexes were visualized using 0.03 % DAB. The sections were examined under a light microscope equipped with a digital camera and scanning program (ZEISS Axio Vert.A1, Carl Zeiss, Göttingen, Germany) [52].

### 2.9 Statistical analysis

Statistical analyses were done on GraphPad Prism® 5.0 (GraphPad Software Inc., San Diego, CA, USA). Differences in body weight and blood glucose were compared using a two-way repeated measures ANOVA. *P* < 0.05 indicates statistically significant differences. If the means of two different groups were statistically significant different, they were analyzed using a post-hoc Bonferroni test. Western blotting analysis, ELISA, and immunohistochemistry data were analyzed using one-way ANOVA and Tukey's post-hoc tests. *, **, and *** indicate *p* < 0.05, <0.01, and <0.001, respectively.

## 3 Results

### 3.1 The effect of resistance exercise on blood glucose levels

**Fig. 1:**
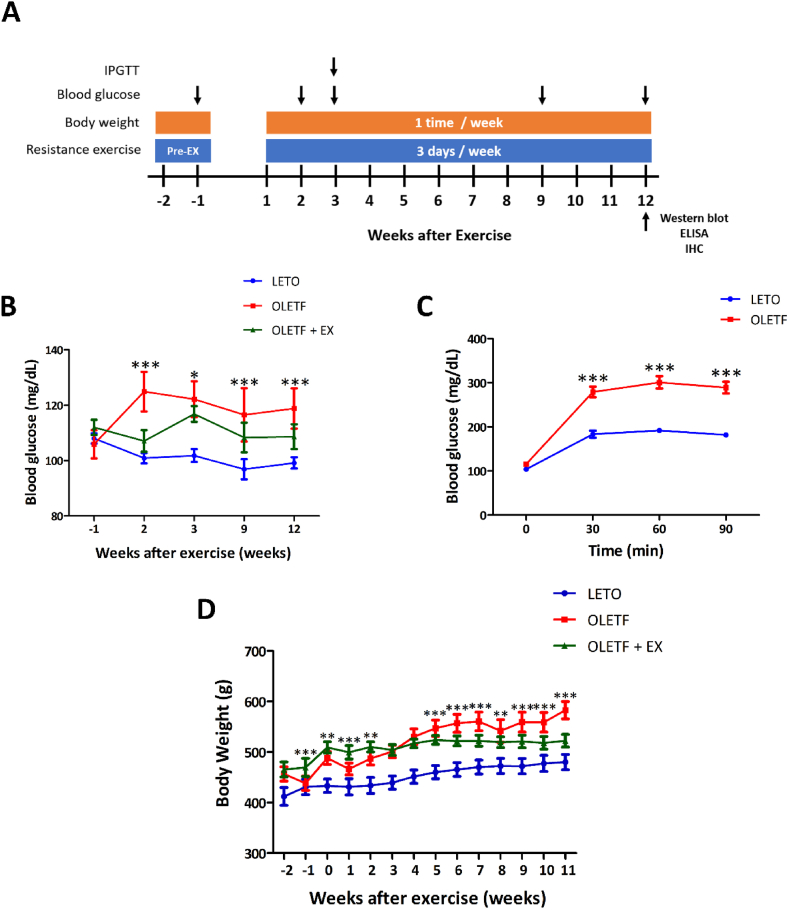
Experimental procedure and a level of blood glucose and body weight. (A) The experimental procedure was conducted according to the following schedule. Pre-exercise was performed before 2 weeks training. Body weight was measured once at every week. Resistance exercise was held three days per week until 12 weeks. At 12 weeks after exercise, tissue was collected for analysis using western blotting, ELISA, and immunohistochemistry. (B) **p < 0.01, ***p < 0.001 compared to the OLETF group. The level of blood glucose of OLETF group was higher than the OLETF + EX group. (C) To assess diabetes induction, the result of an IPGTT was examined. The IPGTT results showed that the glucose values from 30 to 90 min after glucose loading were significantly lower in the LETO group than in the OLETF group. (D) **p < 0.01, ***p < 0.001, and ***p < 0.001 compared with OLETF. The body weight of OLETF group was increased compared with the OLETF + EX groups until 11 weeks.

We decided to exercise for the study and all the experimental procedure was performed following the schedule, which explained in Fig. 1A. For 14 weeks, the animal had a pre-training and regular exercise for three days/week and was checked the body weight and the level of glucose in the blood. After 12 weeks, tissue was collected for the molecular works such as western blotting analysis, ELISA, and immunohistochemistry. To characterize the development of T2DM in OLETF, we monitored the rats’ blood glucose levels. This analysis showed that fasting insulin levels were significantly higher in OLETF rats when compared with LETO rats. Moreover, blood glucose levels were significantly lower in the OLETF + EX group when compared with the OLETF group at weeks 2, 9, and 12 (Fig. 1B). In addition, the IPGTT method confirmed that glucose levels after 30, 60, and 90 min were continuously sustained in the OLETF group when compared with the LETO group (Fig. 1C).

### 3.2 The effect of resistance exercise on body weight

During the experiment, body weight in the OLETF group was consistently higher than in the LETO group. The OLETF group's body weight increased slightly from the beginning to the end of the experiment. However, the OLETF + EX group did not exhibit a significant difference in the body weight at the start versus the end of the experiments. Five weeks after exercise, the OLETF + EX group had a lower body weight than the OLETF group, which persisted until the end of the exercise (Fig. 1D).

### 3.3 The effect of exercise on cardiovascular factors and inflammatory markers

**Fig. 2:**
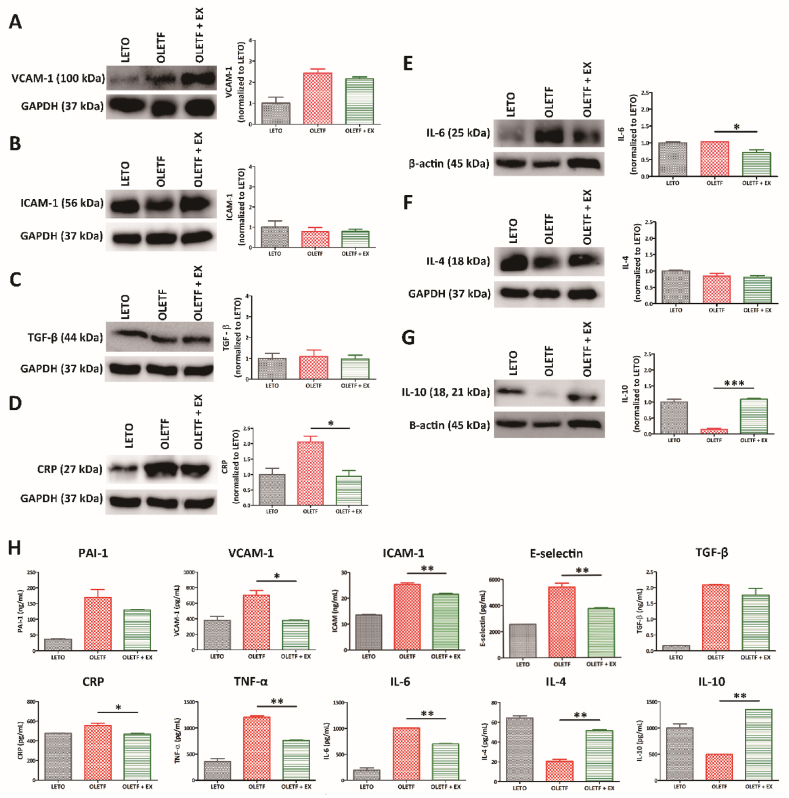
Expression of cardiovascular factors and inflammatory markers. The levels of cardiovascular factors, VACM-1(A) and ICAM-1 (B), fibrotic marker, TGFβ (C), cardiovascular inflammatory marker, CRP (D), pro-inflammatory marker, IL-6 (E), and anti-inflammatory markers, IL-4 (F) and IL-10 (G) were determined via western blotting analysis. GAPDH and β-actin were used as an endogenous control. (H) The protein levels of cardiovascular factors (PAI-1, VACM-1, ICAM-1 and E-selectin), fibrotic marker (TGF-β), cardiovascular inflammatory marker (CRP), pro-inflammatory markers, (TNF-α and IL-6), and anti-inflammatory markers, (IL-4 and IL-10) were determined via ELISA assay. All experiments were repeated at least three times. *p < 0.05, ***p < 0.001 compared with the OLETF group.

We used western blotting analysis to assess the levels of cardiovascular factors and inflammatory markers, including VCAM-1, ICAM-1, TGF-β, CRP, IL-6, IL-4, and IL-10 (Fig. 2 and Fig. S1). This analysis revealed that the exercise group expressed lower levels of the cardiovascular inflammation and pro-inflammatory markers, IL-6 and CRP when compared with the OLETF group (Fig. 2D and E). However, the level of IL-10, an anti-inflammatory marker, was decreased in the OLETF group and increased in the resistance exercise group (Fig. 2G). The levels of the cardiovascular factors did not differ significantly between the OLETF and OLETF + EX groups (Fig. 2A–C). In addition, we did not obtain results from the western blotting analysis of the expression of PAI-1, E-selectin, and TNF-α (data not shown). Furthermore, there were no significant differences in the levels of the anti-inflammatory marker, IL-4. These results indicate that resistance exercise reduces inflammation by regulating pro-inflammatory markers in the rat model of diabetes.

Validation of the western blotting analysis using ELISA revealed lower levels of VCAM-1, ICAM-1, E-selectin, CRP, IL-6, and TNF- α in the OLETF + EX group when compared with the OLETF group (Fig. 2H**)**. Interestingly, the anti-inflammatory markers, IL-4 and IL-10, were highly expressed after exercise. Taken together, these data show that exercise influences the regulation of inflammation by upregulating anti-inflammatory factors and the downregulating cardiovascular factors and pro-inflammatory markers.

### 3.4 The effect of resistance exercise on cardiovascular factors in the heart

**Fig. 3:**
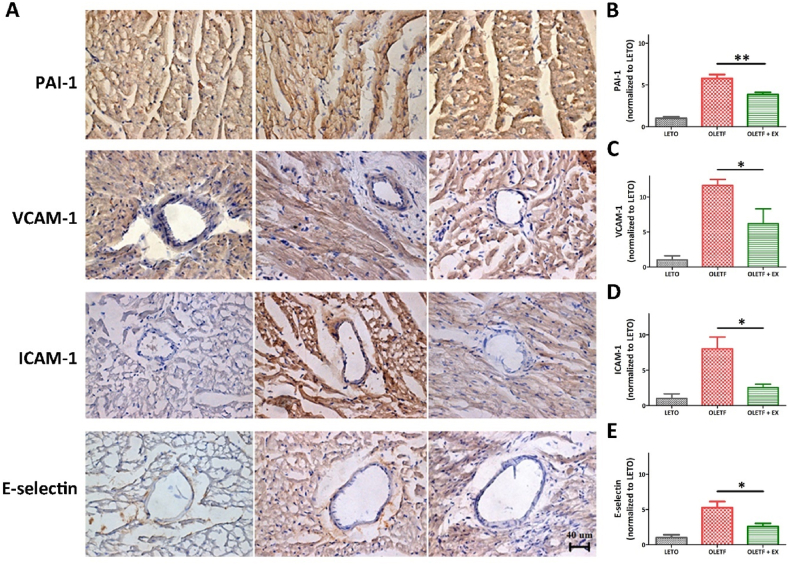
Immunohistochemical assay for cardiovascular factor. The cardiovascular factor was detected by DAB staining (A) and the quantification of the stained area (B–E). The scale bar indicated 40 μm. All experiments were repeated at least three times. *p < 0.05, **p < 0.01 compared to OLETF group.

To validate the observations mad using western blotting analysis and ELISA, we assessed the levels of the cardiovascular factors and inflammatory markers in the rats’ heart using immunohistochemistry. The thrombosis and atherosclerosis risk factor, PAI-1, was detectable in the hearts of the LETO and OLETF groups, but its intensity was significantly higher in the OLETF group when compared with OLETF + EX group (Fig. 3A and B). The endothelial markers, VCAM-1, ICAM-1, and E-selectin, were detected in the vicinity of cardiac vessels. Notably, the staining intensity was significantly higher in the OLETF group when compared with the OLETF + EX group (Fig. 3A and 3C–E). This analysis revealed that PAI-1, VACM-1, ICAM-1, and E-selectin were significantly decreased after exercise.

**Fig. 4:**
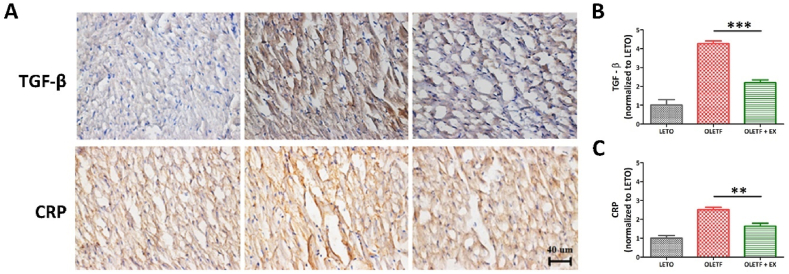
Immunohistochemical assay for fibrotic and cardiovascular inflammatory factor. The fibrotic and cardiovascular inflammatory factor was detected by DAB staining (A) and the quantification of the stained area (B and C). The scale bar indicated 40 μm **p < 0.01, ***p < 0.001 compared to OLETF group.

The cardiovascular inflammatory factor, CRP, was detected in the LETO and OLETF groups, but its intensity was significantly higher in the OLETF group when compared with the OLETF + EX group (Fig. 4A and C). The fibrotic factor, TGF-β, was markedly higher in the OLETF group when compared with the OLETF + EX group (Fig. 4A–B). Taken together, fibrotic and cardiovascular inflammatory factors were evaluated via immunohistochemical analysis of TGF-β and CRP expression. These findings suggest that exercise reduced the levels of diabetes-associated cardiovascular risk factors.

### 3.5 The effect of resistance exercise on inflammatory markers in the heart

**Fig. 5:**
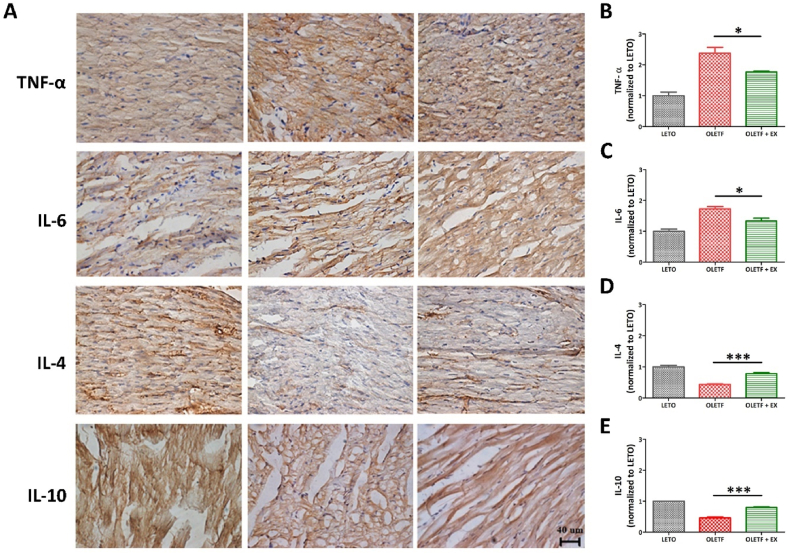
Immunohistochemical assay inflammatory factor. The inflammatory factor was detected by DAB staining (A) and the quantification of the stained area (B–E). The scale bar indicated 40 μm *p < 0.05, ***p < 0.001 compared to OLETF group.

While the pro-inflammatory markers, TNF-α and IL-6, were detectable in the LETO and OLETF groups, their staining intensity was markedly higher in the OLETF group when compared with the OLETF + EX group (Fig. 5A–C). Moreover, the expression of the anti-inflammatory markers, IL-4 and IL-10, was significantly higher in the OLETF + EX group when compared with the OLETF group (Fig. 5A and 5D–E).

## 4 Discussion

In this study, diabetic rats underwent resistance training (three times/week and eight reps/session) to reduce inflammation and improve cardiovascular function. Our analysis revealed that resistance exercise gradually reduced the rats’ body weight and glucose levels. Moreover, it reduced the levels of the cardiovascular factors, PAI-1, VACM-1, ICAM-1, and E-selectin, the cardiovascular inflammatory marker, CRP, the fibrotic marker, TGF-β, and the pro-inflammatory markers, TNF-α and IL-6. Interestingly, western blotting analysis, ELISA, and immunohistochemistry revealed that the exercise group had increased levels of the anti-inflammatory markers, IL-4 and IL-10, rather than the non-exercise group. These results indicate that resistance exercise may help patients with diabetes more than other exercise and that these effects occur through the regulation of cardiovascular factors and inflammation.

The latest evidence shows that diabetes continues to be a large and increasing global health burden and is likely to continue to grow substantially through changing the lifestyle in the next decades [53]. The prevalence of T2DM is gradually increasing worldwide and is projected to reach 700 million cases by 2045 [54]. Without additional intervention, its mortality will continue to increase. The increasing diabetes prevalence is associated with obesity and fatty acid release, reduced insulin levels in muscles, and fat accumulation in liver, which disrupts glucose homeostasis [[55], [56], [57]]. Diabetes is characterized by symptoms of high blood glucose levels, including increased thirst and hunger, and frequent urination [58]. Diabetes is also associated with a 2–4-fold increase in the risk of myocardial ischemia-associated heart failure [59]. T2DM is the consequence of insufficient insulin secretion relative to insulin resistance with advanced age, excessive weight gain, and insufficient physical activity. Among genomic regions in T2DM, several genes are associated in regulation of insulin processing and secretion [[60], [61], [62], [63]].

To treat diabetes, many studies and clinicians have focused on maintaining healthy diet, daily medication, and regular physical exercise [58,64]. When diet and exercise fail to control weight loss and hyperglycemia, patients need antidiabetic agents, such as metformin, which is obtained from natural sources, such as herbs [65]. For drug treatment, the anti-diabetic drugs are mainly comprised of insulin, insulin analogs, and non-insulin hypoglycemic drugs [[66], [67], [68]]. Surgery is sometimes very effective in patients with both obesity and diabetes [58,69]. In addition, recent research showed that a bariatric surgery for treatment of T2DM in people with obesity led to superior glycemic control compared with medical or lifestyle intervention for a long-term management [70]. However, the side effect of bariatric surgery, such as chronic kidney disease, is important, so that the combination of medication and lifestyle is considered more effective and less consuming than surgical treatment [71].

Since the early 1900's, there has been interest in understanding the physiology of exercise in helping people with diabetes to regulate blood glucose and improve insulin sensitivity [72]. It was recognized in ancient times that physical exercise may be beneficial against diabetes [73]. The American Diabetes Association recommends that people with diabetes engage in aerobic activity for at least 150 min each week, and two to three sessions of resistance training exercise per week [74]. Aerobic training increases mitochondrial density, insulin sensitivity, oxidative enzyme levels, lung function, immune function, and cardiac output [75,76]. After exercise, insulin activity in the muscle and liver is enhanced, thereby improving glucose uptake. Resistance training reduced the glucose level in the blood of diabetes even though it was gradually increased in diabetes without exercise by time-dependent manner (Fig. 1).

In the clinic, aerobic exercise is beneficial for glycemic control and weight loss in patients with diabetes [77]. However, research on resistance training in patients with T2DM, both dry-bench and wet-bench, is limited. Although individual exercise studies in patients have shown partial results, exercise is expected to reduce the risk of diabetes [77]. In diabetic patients, before starting exercise sessions, must be carefully educated about the consequences of physical activity on their blood glucose and the appropriate modifications of diet and insulin therapy [78]. Long-term regular exercise is particularly advantageous for T2DM to reduce visceral fat mass and body weight without decreasing lean body mass [79]. In addition, regular aerobic exercise could ameliorate insulin sensitivity, glucose and blood pressure control, lipid profile and reduce the cardiovascular risk [80,81]. In our study, resistance exercise enhanced the level of cardiovascular factors in diabetes animal (Fig. 2, Fig. 3). In addition, the cardiovascular function could be recovered by reducing of fibrosis and cardiovascular inflammation (Fig. 4). The association between T2DM and heart failure remains inconclusive about reduced peripheral oxygen extraction, impaired heart rate adjustment, and lower anaerobic threshold, compared to non-diabetic subjects [82]. However, our results showed the potential of exercise through regulating of cardiovascular factors in diabetes patients, especially resistance exercise by occasionally. In addition, the understanding of cardiopulmonary response would allow the early identification at a higher risk of developing and help to understand the pathophysiological connection between T2DM and heart failure [82].

Lower level of inflammation contributes to the occurrence of metabolic syndrome. The markers of inflammation are pro-inflammatory cytokines/chemokines, such as IL-1β, IL-6, TNF-α, NF-κB, and interferon-γ [83,84]. Resistance training relates to a reduction in inflammatory markers in patients with T2DM, since those patients have reduced activation of anti-inflammatory pathway in a long-term period [85,86]. Inflammation is triggered by the innated immune system and act by surveillance of pathogens via recognition of receptors. The phenotypes of inflammation such as pro- and anti-inflammation have function differently following tissues including muscles, adipose tissues, vessels or etc. Exercise-induced effects on inflammation have additional complexity by specific pro-inflammatory cytokines. Early sport science studies revealed an increase in IL-6 by prolonged endurance exercise. After then, it was resulted of muscle damage and subsequent inflammation. Resistance exercise reduced the pro-inflammation and enhanced the anti-inflammation (Fig. 5). The time-course of resistance training-induced adaptations on the endothelium is still not clear, even though vascular alterations may occur from the first week of exercise and be overcome by later adaptations [87].

In recent years, diabetes treatments, which not only lower blood glucose levels but decrease cardiovascular dysfunction, have been shown to reduce major cardiovascular events [88]. However, those cellular mechanisms are not fully explaining the key factors in diabetes treatment. An in-depth understanding of the signaling pathways and mechanisms involved in exercise's benefits for cardiovascular health will guide the development of novel therapeutic targets and strategies in the future [89]. Several studies already confirmed that the aerobic exercise could improve the cardiovascular function. The most important factor is resistance exercise, which has beneficial effects, such as enhancing cardiovascular factors and reducing inflammation. Therefore, we suggest a continuous resistance training program for people with diabetes.

Resistance exercise that requires lower cardiorespiratory fitness than aerobic exercise has a beneficial effect on metabolic disease patients who are not able to perform aerobic exercises for a long time. Although we suggested the positive potential of exercise, the limitations of our work still remain. Firstly, we investigated the effect of the resistance training not an aerobic exercise in the in vivo diabetes model. Second, most of T2DM patients are old-aged, however, we did not consider to make a training program for the aged person. Third, recent studies identified the exercise-regulated molecules as potential biomarkers for guiding a more effective and safe exercise training for patients; however, we did not check the effect of them yet. Therefore, to find a regulator of diabetes and to apply the suitable training for subject is our next goal for T2DM therapy, such as miRNAs. Despite the rapid and cost-effective development, the gene therapy needs further exploration of lower immunogenicity.

## 5 Conclusions

We revealed that the resistance exercise gradually reduced the body weight and glucose level in the diabetic rats. Moreover, exercise has a potential effect to reduce the inflammation and induce the cardiovascular function. Following the results, the resistance training for people with diabetes might be a key factor to rescue the disease.

## Data availability statement

All data generated or analyzed during this study are included in this published article and supplementary information.

## CRediT authorship contribution statement

**Jin Yoo:** Investigation, Funding acquisition, Data curation, Conceptualization. **Jinsu Hwang:** Software, Formal analysis, Data curation. **Jiyun Choi:** Formal analysis, Data curation. **Mahesh Ramalingam:** Validation, Software, Formal analysis, Data curation. **Haewon Jeong:** Formal analysis, Data curation. **Sujeong Jang:** Supervision, Software, Investigation, Funding acquisition, Formal analysis, Data curation, Conceptualization. **Han-Seong Jeong:** Supervision, Project administration, Investigation, Funding acquisition. **Daeyeol Kim:** Supervision, Project administration, Investigation, Funding acquisition, Conceptualization.

## Declaration of competing interest

The authors declare the following financial interests/personal relationships which may be considered as potential competing interests:Sujeong Jang, Han-Seong Jeong, Jin Yoo reports equipment, drugs, or supplies was provided by National Research Foundation of Korea. Han-Seong Jeong reports article publishing charges and equipment, drugs, or supplies were provided by Korea Institute for Advancement of Technology. If there are other authors, they declare that they have no known competing financial interests or personal relationships that could have appeared to influence the work reported in this paper.

## References

[1] Galtier F. (2010). Definition, epidemiology, risk factors. Diabetes Metab..

[2] American Diabetes A. (2014). Diagnosis and classification of diabetes mellitus. Diabetes Care.

[3] Kharroubi A.T., Darwish H.M. (2015). Diabetes mellitus: the epidemic of the century. World J. Diabetes.

[4] Qaid M.M., Abdelrahman M.M. (2016). Role of insulin and other related hormones in energy metabolism—a review. Cogent Food Agric..

[5] Kakleas K., Soldatou A., Karachaliou F., Karavanaki K. (2015). Associated autoimmune diseases in children and adolescents with type 1 diabetes mellitus (T1DM). Autoimmun. Rev..

[6] Katsarou A., Gudbjornsdottir S., Rawshani A., Dabelea D., Bonifacio E., Anderson B.J., Jacobsen L.M., Schatz D.A., Lernmark A. (2017). Type 1 diabetes mellitus. Nat. Rev. Dis. Prim..

[7] Paschou S.A., Papadopoulou-Marketou N., Chrousos G.P., Kanaka-Gantenbein C. (2018). On type 1 diabetes mellitus pathogenesis. Endocr Connect.

[8] Ilonen J., Lempainen J., Veijola R. (2019). The heterogeneous pathogenesis of type 1 diabetes mellitus. Nat. Rev. Endocrinol..

[9] Akash M.S., Rehman K., Chen S. (2013). Role of inflammatory mechanisms in pathogenesis of type 2 diabetes mellitus. J. Cell. Biochem..

[10] Chao M.L., Luo S., Zhang C., Zhou X., Zhou M., Wang J., Kong C., Chen J., Lin Z., Tang X., Sun S., Tang X., Chen H., Wang H., Wang D., Sun J.P., Han Y., Xie L., Ji Y. (2021). S-nitrosylation-mediated coupling of G-protein alpha-2 with CXCR5 induces Hippo/YAP-dependent diabetes-accelerated atherosclerosis. Nat Commun.

[11] Einarson T.R., Acs A., Ludwig C., Panton U.H. (2018). Prevalence of cardiovascular disease in type 2 diabetes: a systematic literature review of scientific evidence from across the world in 2007-2017. Cardiovasc. Diabetol..

[12] Emerging Risk Factors C., Sarwar N., Gao P., Seshasai S.R., Gobin R., Kaptoge S., Di Angelantonio E., Ingelsson E., Lawlor D.A., Selvin E., Stampfer M., Stehouwer C.D., Lewington S., Pennells L., Thompson A., Sattar N., White I.R., Ray K.K., Danesh J. (2010). Diabetes mellitus, fasting blood glucose concentration, and risk of vascular disease: a collaborative meta-analysis of 102 prospective studies. Lancet.

[13] Martin-Timon I., Sevillano-Collantes C., Segura-Galindo A., Del Canizo-Gomez F.J. (2014). Type 2 diabetes and cardiovascular disease: have all risk factors the same strength?. World J. Diabetes.

[14] Akash M.S.H., Rehman K., Liaqat A. (2018). Tumor necrosis factor-alpha: role in development of insulin resistance and pathogenesis of type 2 diabetes mellitus. J. Cell. Biochem..

[15] Rihawi K., Ricci A.D., Rizzo A., Brocchi S., Marasco G., Pastore L.V., Llimpe F.L.R., Golfieri R., Renzulli M. (2021). Tumor-associated macrophages and inflammatory microenvironment in gastric cancer: novel translational implications. Int. J. Mol. Sci..

[16] Vitale E., Rizzo A., Santa K., Jirillo E. (2024). Associations between "cancer risk", "inflammation" and "metabolic syndrome": a scoping review. Biology.

[17] Indio V., Schipani A., Nannini M., Urbini M., Rizzo A., De Leo A., Altimari A., Di Scioscio V., Messelodi D., Tarantino G., Astolfi A., Pantaleo M.A. (2021). Gene expression landscape of SDH-deficient gastrointestinal stromal tumors. J. Clin. Med..

[18] Rizzo A., Santoni M., Mollica V., Fiorentino M., Brandi G., Massari F. (2022). Microbiota and prostate cancer. Semin. Cancer Biol..

[19] Di Federico A., Mosca M., Pagani R., Carloni R., Frega G., De Giglio A., Rizzo A., Ricci D., Tavolari S., Di Marco M., Palloni A., Brandi G. (2022). Immunotherapy in pancreatic cancer: why do we keep failing? A focus on tumor immune microenvironment, predictive biomarkers and treatment outcomes. Cancers.

[20] Vaughan D.E. (2005). PAI-1 and atherothrombosis. J Thromb Haemost.

[21] Ploplis V.A. (2011). Effects of altered plasminogen activator inhibitor-1 expression on cardiovascular disease. Curr. Drug Targets.

[22] Ghosh A.K., Vaughan D.E. (2012). PAI-1 in tissue fibrosis. J. Cell. Physiol..

[23] Baluta M.M., Vintila M.M. (2015). PAI-1 inhibition - another therapeutic option for cardiovascular protection. Maedica (Bucur).

[24] Schneider D.J., Sobel B.E. (2012). PAI-1 and diabetes: a journey from the bench to the bedside. Diabetes Care.

[25] Van De Craen B., Declerck P.J., Gils A. (2012). The Biochemistry, Physiology and Pathological roles of PAI-1 and the requirements for PAI-1 inhibition in vivo. Thromb. Res..

[26] Martins S.R., Toledo S.L.O., da Silva A.J., Mendes F.S., de Oliveira M.M., Ferreira L.G.R., Dusse L.M.S., Carvalho M.D.G., Rios D.R.A., Alpoim P.N., Pinheiro M.B. (2022). Endothelial dysfunction biomarkers in sickle cell disease: is there a role for ADMA and PAI-1?. Ann. Hematol..

[27] Gupta S.K., Dongare S., Mathur R., Mohanty I.R., Srivastava S., Mathur S., Nag T.C. (2015). Genistein ameliorates cardiac inflammation and oxidative stress in streptozotocin-induced diabetic cardiomyopathy in rats. Mol. Cell. Biochem..

[28] Knudsen S.H., Pedersen B.K. (2015). Targeting inflammation through a physical active lifestyle and pharmaceuticals for the treatment of type 2 diabetes. Curr Diab Rep.

[29] Karstoft K., Pedersen B.K. (2016). Exercise and type 2 diabetes: focus on metabolism and inflammation. Immunol. Cell Biol..

[30] Liu C., Feng X., Li Q., Wang Y., Li Q., Hua M. (2016). Adiponectin, TNF-alpha and inflammatory cytokines and risk of type 2 diabetes: a systematic review and meta-analysis. Cytokine.

[31] Lee J., Lee S., Zhang H., Hill M.A., Zhang C., Park Y. (2017). Interaction of IL-6 and TNF-alpha contributes to endothelial dysfunction in type 2 diabetic mouse hearts. PLoS One.

[32] Zanuso S., Balducci S., Jimenez A. (2009). Physical activity, a key factor to quality of life in type 2 diabetic patients. Diabetes Metab Res Rev.

[33] Balducci S., Sacchetti M., Haxhi J., Orlando G., D'Errico V., Fallucca S., Menini S., Pugliese G. (2014). Physical exercise as therapy for type 2 diabetes mellitus. Diabetes Metab Res Rev.

[34] Mann S., Beedie C., Balducci S., Zanuso S., Allgrove J., Bertiato F., Jimenez A. (2014). Changes in insulin sensitivity in response to different modalities of exercise: a review of the evidence. Diabetes Metab Res Rev.

[35] Pesta D.H., Goncalves R.L.S., Madiraju A.K., Strasser B., Sparks L.M. (2017). Resistance training to improve type 2 diabetes: working toward a prescription for the future. Nutr. Metab..

[36] Sabouri M., Hatami E., Pournemati P., Shabkhiz F. (2021). Inflammatory, antioxidant and glycemic status to different mode of high-intensity training in type 2 diabetes mellitus. Mol. Biol. Rep..

[37] Jayedi A., Emadi A., Shab-Bidar S. (2022). Dose-dependent effect of supervised aerobic exercise on HbA(1c) in patients with type 2 diabetes: a meta-analysis of randomized controlled trials. Sports Med..

[38] Eves N.D., Plotnikoff R.C. (2006). Resistance training and type 2 diabetes: considerations for implementation at the population level. Diabetes Care.

[39] Ciccolo J.T., Carr L.J., Krupel K.L., Longval J.L. (2010). The role of resistance training in the prevention and treatment of chronic disease. Am. J. Lifestyle Med..

[40] Hall K.E., McDonald M.W., Grise K.N., Campos O.A., Noble E.G., Melling C.W. (2013). The role of resistance and aerobic exercise training on insulin sensitivity measures in STZ-induced Type 1 diabetic rodents. Metabolism.

[41] Flack K.D., Davy K.P., Hulver M.W., Winett R.A., Frisard M.I., Davy B.M. (2010). Aging, resistance training, and diabetes prevention. J Aging Res.

[42] Westcott W.L. (2012). Resistance training is medicine: effects of strength training on health. Curr. Sports Med. Rep..

[43] Abd El-Kader S.M. (2011). Aerobic versus resistance exercise training in modulation of insulin resistance, adipocytokines and inflammatory cytokine levels in obese type 2 diabetic patients. J. Adv. Res..

[44] Calle M.C., Fernandez M.L. (2010). Effects of resistance training on the inflammatory response. Nutr Res Pract.

[45] Hemanthakumar K.A., Fang S., Anisimov A., Mayranpaa M.I., Mervaala E., Kivela R. (2021). Cardiovascular disease risk factors induce mesenchymal features and senescence in mouse cardiac endothelial cells. Elife.

[46] Kwon H.R., Min K.W., Ahn H.J., Seok H.G., Lee J.H., Park G.S., Han K.A. (2011). Effects of aerobic exercise vs. Resistance training on endothelial function in women with type 2 diabetes mellitus. Diabetes Metab. J.

[47] Shimomura M., Horii N., Fujie S., Inoue K., Hasegawa N., Iemitsu K., Uchida M., Iemitsu M. (2021). Decreased muscle-derived musclin by chronic resistance exercise is associated with improved insulin resistance in rats with type 2 diabetes. Physiol Rep.

[48] Dinger K., Mohr J., Vohlen C., Hirani D., Hucklenbruch-Rother E., Ensenauer R., Dotsch J., Alejandre Alcazar M.A. (2018). Intraperitoneal glucose tolerance test, measurement of lung function, and fixation of the lung to study the impact of obesity and impaired metabolism on pulmonary outcomes. J. Vis. Exp..

[49] Soni K.K., Hwang J., Ramalingam M., Kim C., Kim B.C., Jeong H.S., Jang S. (2023). Endoplasmic reticulum stress causing apoptosis in a mouse model of an ischemic spinal cord injury. Int. J. Mol. Sci..

[50] Ramalingam M., Jang S., Jeong H.S. (2021). Therapeutic effects of conditioned medium of neural differentiated human bone marrow-derived stem cells on rotenone-induced alpha-synuclein aggregation and apoptosis. Stem Cells Int.

[51] Karamese M., Aydin H., Sengul E., Gelen V., Sevim C., Ustek D., Karakus E. (2016). The immunostimulatory effect of lactic acid bacteria in a rat model. Iran J Immunol.

[52] Jang S., Cho H.H., Kim S.H., Lee K.H., Cho Y.B., Park J.S., Jeong H.S. (2016). Transplantation of human adipose tissue-derived stem cells for repair of injured spiral ganglion neurons in deaf Guinea pigs. Neural Regen Res.

[53] Guariguata L., Whiting D.R., Hambleton I., Beagley J., Linnenkamp U., Shaw J.E. (2014). Global estimates of diabetes prevalence for 2013 and projections for 2035. Diabetes Res. Clin. Pract..

[54] Magkos F., Hjorth M.F., Astrup A. (2020). Diet and exercise in the prevention and treatment of type 2 diabetes mellitus. Nat. Rev. Endocrinol..

[55] Mittendorfer B., Magkos F., Fabbrini E., Mohammed B.S., Klein S. (2009). Relationship between body fat mass and free fatty acid kinetics in men and women. Obesity.

[56] Conte C., Fabbrini E., Kars M., Mittendorfer B., Patterson B.W., Klein S. (2012). Multiorgan insulin sensitivity in lean and obese subjects. Diabetes Care.

[57] Wilman H.R., Kelly M., Garratt S., Matthews P.M., Milanesi M., Herlihy A., Gyngell M., Neubauer S., Bell J.D., Banerjee R., Thomas E.L. (2017). Characterisation of liver fat in the UK Biobank cohort. PLoS One.

[58] Bailey C., Skouteris H., Teede H., Hill B., De Courten B., Walker R., Liew D., Thangaratinam S., Ademi Z. (2020). Are lifestyle interventions to reduce excessive gestational weight gain cost effective? A systematic review. Curr Diab Rep.

[59] Park J.J. (2021). Epidemiology, pathophysiology, diagnosis and treatment of heart failure in diabetes. Diabetes Metab. J.

[60] Dimas A.S., Lagou V., Barker A., Knowles J.W., Magi R., Hivert M.F., Benazzo A., Rybin D., Jackson A.U., Stringham H.M., Song C., Fischer-Rosinsky A., Boesgaard T.W., Grarup N., Abbasi F.A., Assimes T.L., Hao K., Yang X., Lecoeur C., Barroso I., Bonnycastle L.L., Bottcher Y., Bumpstead S., Chines P.S., Erdos M.R., Graessler J., Kovacs P., Morken M.A., Narisu N., Payne F., Stancakova A., Swift A.J., Tonjes A., Bornstein S.R., Cauchi S., Froguel P., Meyre D., Schwarz P.E., Haring H.U., Smith U., Boehnke M., Bergman R.N., Collins F.S., Mohlke K.L., Tuomilehto J., Quertemous T., Lind L., Hansen T., Pedersen O., Walker M., Pfeiffer A.F., Spranger J., Stumvoll M., Meigs J.B., Wareham N.J., Kuusisto J., Laakso M., Langenberg C., Dupuis J., Watanabe R.M., Florez J.C., Ingelsson E., McCarthy M.I., Prokopenko I., Investigators M. (2014). Impact of type 2 diabetes susceptibility variants on quantitative glycemic traits reveals mechanistic heterogeneity. Diabetes.

[61] McCarthy M.I. (2010). Genomics, type 2 diabetes, and obesity. N. Engl. J. Med..

[62] Kretowski A., Ruperez F.J., Ciborowski M. (2016). Genomics and metabolomics in obesity and type 2 diabetes. J. Diabetes Res..

[63] Diedisheim M., Carcarino E., Vandiedonck C., Roussel R., Gautier J.F., Venteclef N. (2020). Regulation of inflammation in diabetes: from genetics to epigenomics evidence. Mol Metab.

[64] Simos Y.V., Spyrou K., Patila M., Karouta N., Stamatis H., Gournis D., Dounousi E., Peschos D. (2021). Trends of nanotechnology in type 2 diabetes mellitus treatment. Asian J. Pharm. Sci..

[65] Abu-Odeh A.M., Talib W.H. (2021). Middle East medicinal plants in the treatment of diabetes: a review. Molecules.

[66] Moroder L., Musiol H.J. (2017). Insulin-from its discovery to the industrial synthesis of modern insulin analogues. Angew Chem. Int. Ed. Engl..

[67] El Naggar N., Kalra S. (2017). Switching from biphasic human insulin to premix insulin analogs: a review of the evidence regarding quality of life and adherence to medication in type 2 diabetes mellitus. Adv. Ther..

[68] Zhao R., Lu Z., Yang J., Zhang L., Li Y., Zhang X. (2020). Drug delivery system in the treatment of diabetes mellitus. Front. Bioeng. Biotechnol..

[69] Cash J.C. (2023).

[70] Courcoulas A.P., Patti M.E., Hu B., Arterburn D.E., Simonson D.C., Gourash W.F., Jakicic J.M., Vernon A.H., Beck G.J., Schauer P.R., Kashyap S.R., Aminian A., Cummings D.E., Kirwan J.P. (2024). Long-term outcomes of medical management vs bariatric surgery in type 2 diabetes. JAMA.

[71] Mingrone G., Panunzi S., De Gaetano A., Guidone C., Iaconelli A., Capristo E., Chamseddine G., Bornstein S.R., Rubino F. (2021). Metabolic surgery versus conventional medical therapy in patients with type 2 diabetes: 10-year follow-up of an open-label, single-centre, randomised controlled trial. Lancet.

[72] (1938). Rev VJTAt.

[73] Horton E.S. (1988). Role and management of exercise in diabetes mellitus. Diabetes Care.

[74] Zahalka S.J., Abushamat L.A., Scalzo R.L., Reusch J.E.B., Feingold K.R., Anawalt B., Blackman M.R., Boyce A., Chrousos G., Corpas E., de Herder W.W., Dhatariya K., Dungan K., Hofland J., Kalra S., Kaltsas G., Kapoor N., Koch C., Kopp P., Korbonits M., Kovacs C.S., Kuohung W., Laferrere B., Levy M., McGee E.A., McLachlan R., New M., Purnell J., Sahay R., Shah A.S., Singer F., Sperling M.A., Stratakis C.A., Trence D.L., Wilson D.P. (2000). The Role of Exercise in Diabetes.

[75] Colberg S.R., Sigal R.J., Yardley J.E., Riddell M.C., Dunstan D.W., Dempsey P.C., Horton E.S., Castorino K., Tate D.F. (2016). Physical activity/exercise and diabetes: a position statement of the American diabetes association. Diabetes Care.

[76] Garber C.E., Blissmer B., Deschenes M.R., Franklin B.A., Lamonte M.J., Lee I.M., Nieman D.C., Swain D.P., American College of Sports M. American College of Sports Medicine position stand (2011). Quantity and quality of exercise for developing and maintaining cardiorespiratory, musculoskeletal, and neuromotor fitness in apparently healthy adults: guidance for prescribing exercise. Med. Sci. Sports Exerc..

[77] Boule N.G., Haddad E., Kenny G.P., Wells G.A., Sigal R.J. (2001). Effects of exercise on glycemic control and body mass in type 2 diabetes mellitus: a meta-analysis of controlled clinical trials. JAMA.

[78] Improta-Caria A.C., De Sousa R.A.L., Roever L., Fernandes T., Oliveira E.M., Aras Junior R., Souza B.S.F. (2022). MicroRNAs in type 2 diabetes mellitus: potential role of physical exercise. Rev. Cardiovasc. Med..

[79] Amanat S., Ghahri S., Dianatinasab A., Fararouei M., Dianatinasab M. (2020). Exercise and type 2 diabetes. Adv. Exp. Med. Biol..

[80] De Feo P., Di Loreto C., Ranchelli A., Fatone C., Gambelunghe G., Lucidi P., Santeusanio F. (2006). Exercise and diabetes. Acta Biomed..

[81] Viggers R., Al-Mashhadi Z., Fuglsang-Nielsen R., Gregersen S., Starup-Linde J. (2020). The impact of exercise on bone health in type 2 diabetes mellitus-a systematic review. Curr. Osteoporos. Rep..

[82] Nesti L., Pugliese N.R., Sciuto P., Natali A. (2020). Type 2 diabetes and reduced exercise tolerance: a review of the literature through an integrated physiology approach. Cardiovasc. Diabetol..

[83] Guillemot-Legris O., Muccioli G.G. (2017). Obesity-induced neuroinflammation: beyond the hypothalamus. Trends Neurosci..

[84] Roh H.T., Cho S.Y., So W.Y. (2020). A cross-sectional study evaluating the effects of resistance exercise on inflammation and neurotrophic factors in elderly women with obesity. J. Clin. Med..

[85] Hopps E., Canino B., Caimi G. (2011). Effects of exercise on inflammation markers in type 2 diabetic subjects. Acta Diabetol..

[86] Rech A., Botton C.E., Lopez P., Quincozes-Santos A., Umpierre D., Pinto R.S. (2019). Effects of short-term resistance training on endothelial function and inflammation markers in elderly patients with type 2 diabetes: a randomized controlled trial. Exp. Gerontol..

[87] Tinken T.M., Thijssen D.H., Hopkins N., Dawson E.A., Cable N.T., Green D.J. (2010). Shear stress mediates endothelial adaptations to exercise training in humans. Hypertension.

[88] Suryasa I.W., Rodríguez-Gámez M., Koldoris T. (2021). Health and treatment of diabetes mellitus. Int. J. Health Sci..

[89] Chen H., Chen C., Spanos M., Li G., Lu R., Bei Y., Xiao J. (2022). Exercise training maintains cardiovascular health: signaling pathways involved and potential therapeutics. Signal Transduct Target Ther.

