# Supplementary material for: The effects of resistance training on cardiovascular factors and anti-inflammation in diabetic rats
Source: Heliyon. 2024 Aug 28;10(17):e37081. doi: 10.1016/j.heliyon.2024.e37081 (PMC11407942; doi:10.1016/j.heliyon.2024.e37081)
Supplement: Multimedia component 1 [file mmc1.docx]

Supplementary Information

**The Effects of Resistance Training on Cardiovascular Factors and Anti-Inflammation in Diabetic Rats**


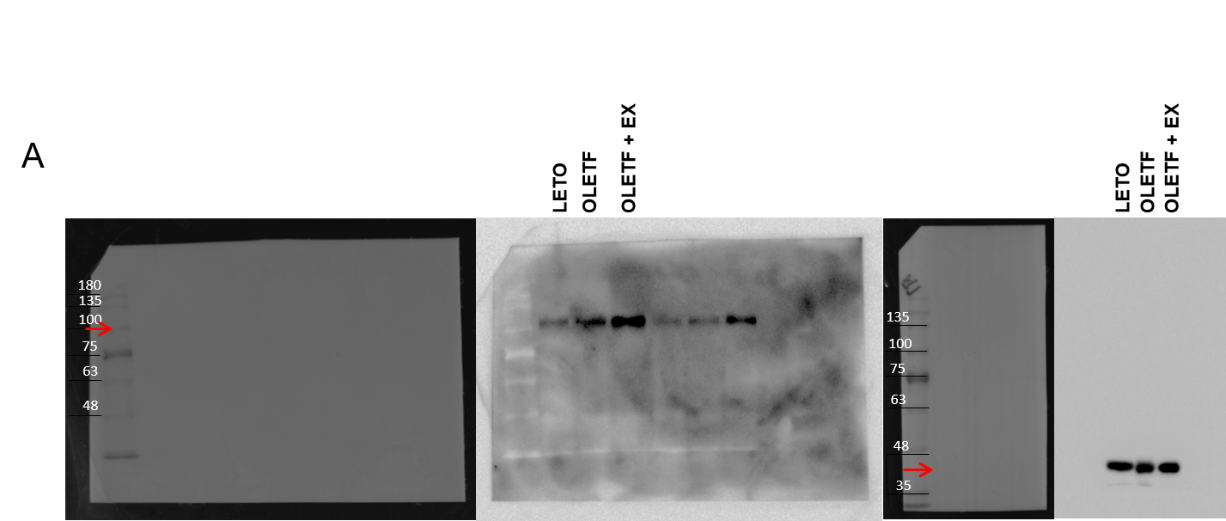

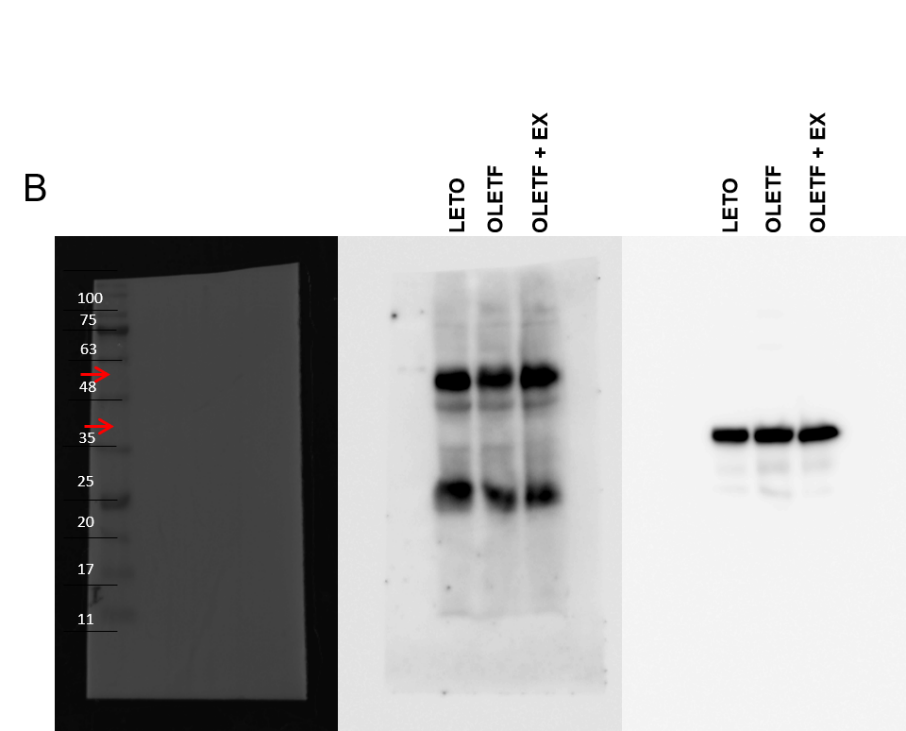


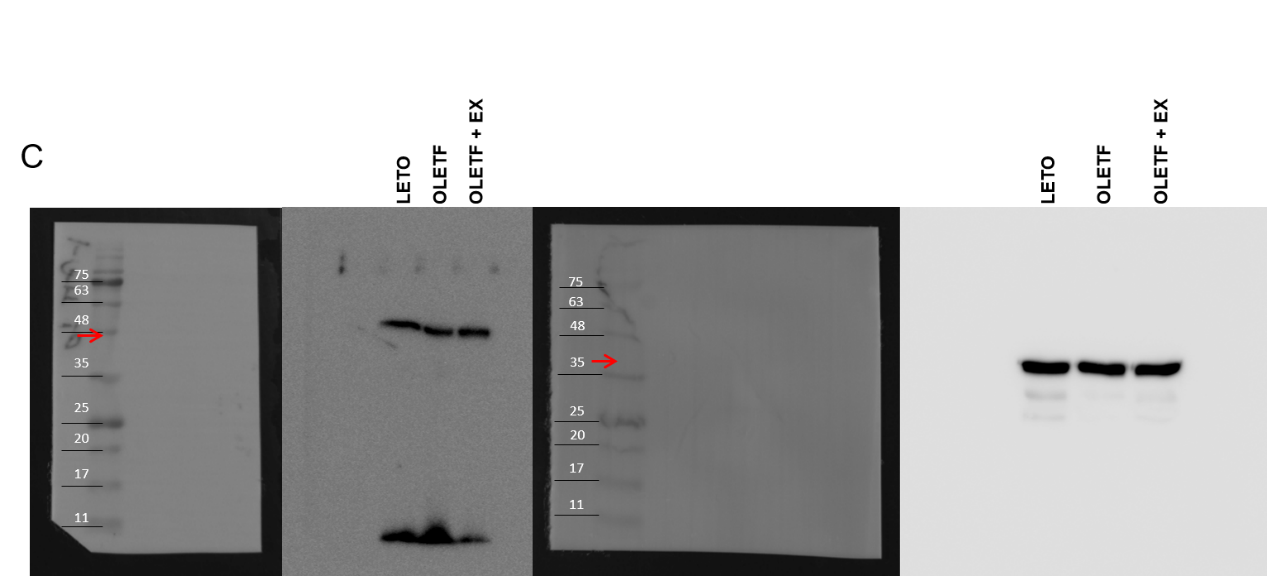


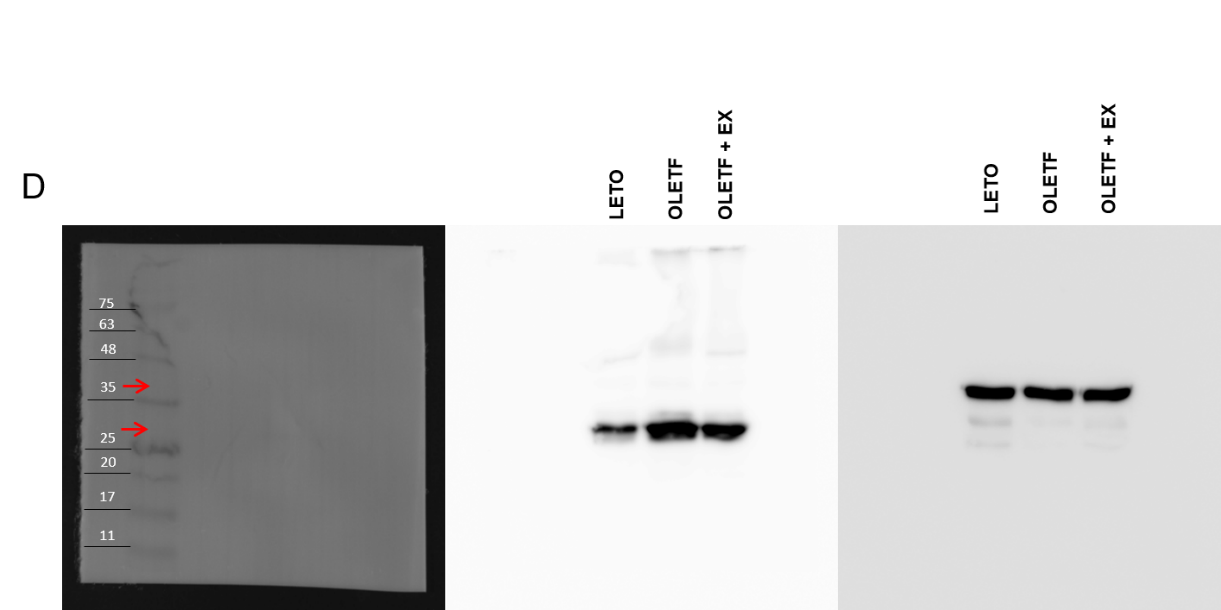


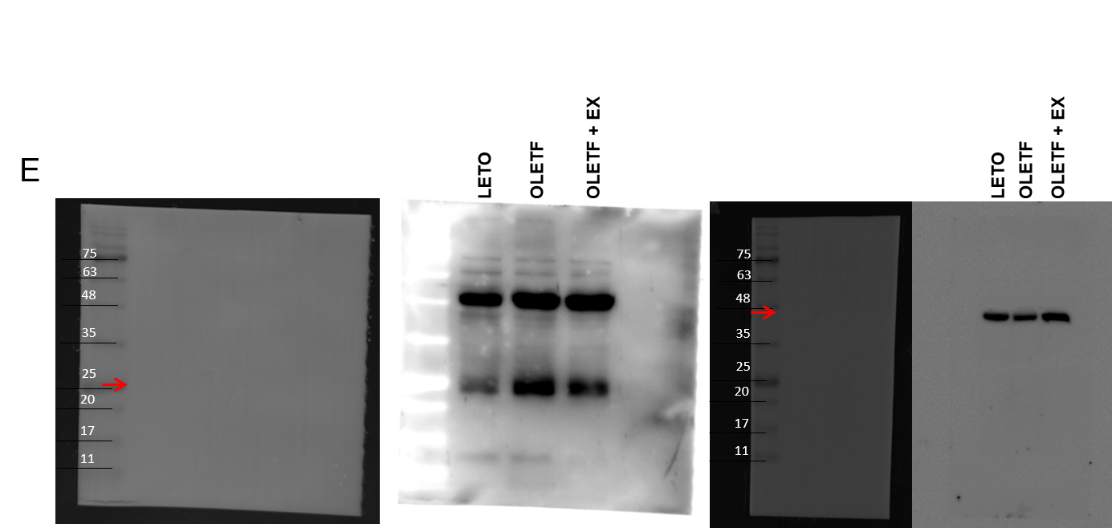


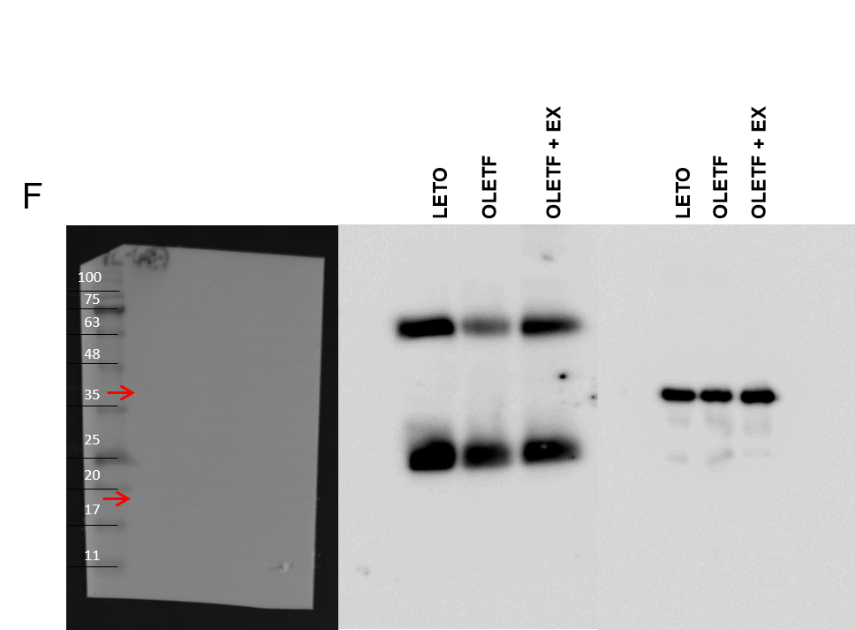


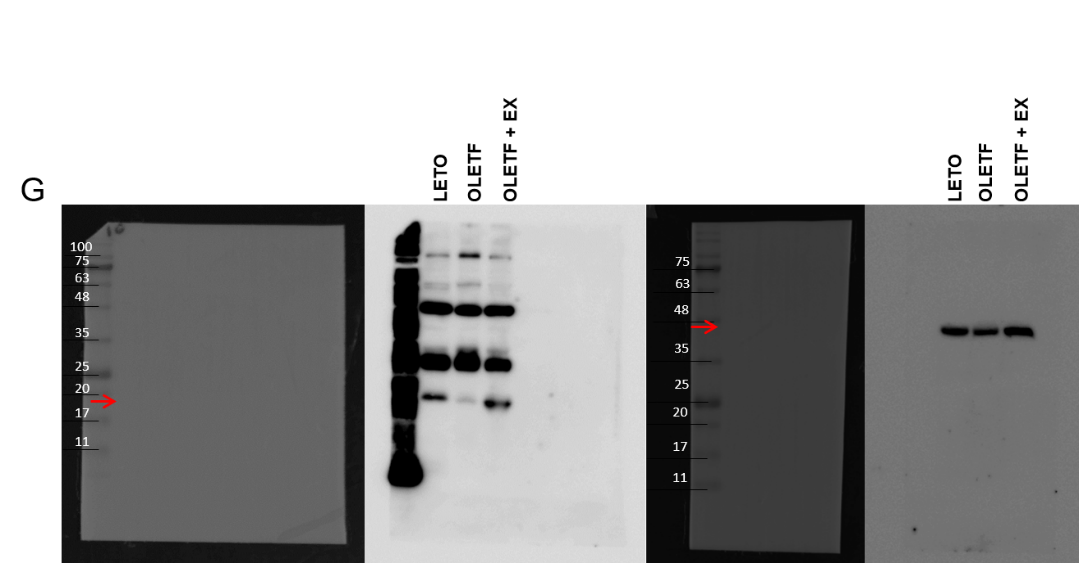


**Fig. S1.** Unedited images and their molecular markers for respective Western blot used in Fig. 2A ~ G of this manuscript.
